# Exploring Self-reported Adherence Measures to Screen for Elevated HIV Viral Load in Adolescents: A South African Cohort Study

**DOI:** 10.1007/s10461-023-04068-2

**Published:** 2023-04-17

**Authors:** Siyanai Zhou, Elona Toska, Nontokozo Langwenya, Olanrewaju Edun, Lucie Cluver, Lucia Knight

**Affiliations:** 1 Division of Social and Behavioural Sciences, School of Public Health, Faculty of Health Sciences, University of Cape Town, Rondebosch, Cape Town, 7700 South Africa; 2 Centre for Social Science Research, University of Cape Town, Cape Town, South Africa; 3 Department of Sociology, University of Cape Town, Cape Town, South Africa; 4 Department of Social Policy and Intervention, University of Oxford, Oxford, UK; 5 MRC Centre for Global Infectious Disease Analysis, School of Public Health, Imperial College London, London, UK; 6 Department of Child and Adolescent Psychiatry, University of Cape Town, Cape Town, South Africa; 7 School of Public Health, University of the Western Cape, Bellville, South Africa

**Keywords:** Adolescents, Self-reported Adherence, Elevated Viral Load, Longitudinal, South Africa

## Abstract

The timely identification of ART non-adherence among adolescents living with HIV presents a significant challenge, particularly in resource-limited settings where virologic monitoring is suboptimal. Using South African adolescent cohort data (N = 933, mean age 13.6 ± 2.89 years, 55.1% female, follow-up = 2014–2018), we examined the association between elevated viral load (VL ≥ 1000 copies/mL) and seven self-reported adherence measures on missed doses, and clinic appointments –with varying recall timeframes. The best performing measures, which were significantly associated with elevated viral load in covariate-adjusted models are: *any missed dose –past 3 days* (sensitivity = 91.6% [95%CI: 90.3–92.8], positive predictive value (PPV) = 78.8% [95%CI: 77.2–80.4]), *–past week* (sensitivity = 87% [95%CI: 85.4–88.6], PPV = 78.2% [95%CI: 76.5–79.9]), *–past month* (sensitivity = 79.5% [95%CI: 77.5–81.4], PPV = 78.2% [95%CI: 76.4–79.9]), *any past-month days missed* (sensitivity = 86.7% [95%CI: 85.1–88.3], PPV = 77.9% [95%CI:76.2–79.6]), and *any missed clinic appointment* (sensitivity = 88.3% [95%CI: 86.8–89.8], PPV = 78.4% [95%CI: 76.8–79.9]). Combining the three best performing measures *missed dose –past 3 days, –past week*, and *any past-year missed clinic appointment* increased sensitivity to 96.4% while maintaining a PPV of about 78%. The discriminatory power of simple and easy-to-administer self-reported adherence measures in detecting elevated viral load warrants consideration in resource-limited settings and may contribute to the aims of the new Global Alliance to End AIDS in children and adolescents by 2030.

**Supplementary Information:**

The online version contains supplementary material available at 10.1007/s10461-023-04068-2.

## Introduction

Scaling up access to antiretroviral treatment (ART) has led to global reductions in HIV-related morbidity and mortality as well as reduced risk of onward HIV transmission [1, 2]. However, for adolescents living with HIV (ALHIV) in sub-Saharan Africa (SSA), the benefits of ART use are yet to be maximised. Adolescents continue to experience life-threatening health vulnerabilities that negatively impact their well-being and survival [3]. Long-term ART adherence among ALHIV remains suboptimal, and lower compared to both younger children and adults [4–6]. For example, studies show that ALHIV are approximately 50% less likely than adults to maintain adherence [7, 8]. ALHIV are more than twice as likely to be lost-to-follow-up than adults [9], with HIV being the fourth leading cause of adolescent deaths in 2015 [10]. In South Africa, adolescents account for the largest share of new HIV infections, and over 421 100 adolescents were estimated to be living with HIV in 2021 [11, 12]. Therefore, more concerted efforts aimed at monitoring and improving long-term ART adherence among ALHIV are urgently needed [13].

While the World Health Organization (WHO) recommends VL monitoring as the gold standard for monitoring HIV treatment success, several challenges exist in making this possible for the majority of countries in SSA [14]. These challenges include human resources (i.e., staffing shortages) and delays in the development of a skilled workforce; weaknesses in sample transport and laboratory workflow; poor laboratory equipment maintenance; and budget limitations [15]. Consequently, VL testing is often infrequent and poorly accessible [16]. Each of these may further delay early identification of non-adherence among ALHIV and subsequently delay interventions to support adherence and regimen switching. Therefore, alternative measures of ART adherence, such as self-reports are essential, in addition to VL monitoring. Evaluating alternative measures of adherence may facilitate understanding of adolescent medication-taking behaviour, a goal for researchers and clinicians [17].

Self-reported ART adherence measures are widely adopted in both clinical practice and research. They are often the only practical and readily available method in resource-limited settings due to their low cost, minimal patient burden, and ease of administration [18, 19]. However, there is limited evidence assessing the validity and consistency of these measures among ALHIV using longitudinal data [20]. Most tools routinely used to measure self-reported adherence have been designed for primarily use in adult populations, and very few studies have assessed the performance of these measures with adolescents [1, 21]. A study in Zimbabwe (N = 173) assessed self-reported adherence measures among older children and adolescents at 48 weeks post-ART initiation. This study found that when patients reported non-adherence measured with items reflecting missed doses in the past three days and weekends, and not taking ART medicine for two days or more in the past three months, it is a strong indicator of a high HIV viral load [22]. Similarly, another study in Cameroon (N = 455) using a single self-reported item on missed doses in the past month found that self-reported adherence was associated with VL [23]. Despite these results, there is a need for more considerations to validate and adapt self-reported measures of adherence to adolescents in order to obtain more accurate measures of ART adherence. Using longitudinal data, our study compared the performance of seven self-reported ART adherence measures with varying recall timeframes and differing missed dose reporting structures in predicting elevated VL. We also assessed the association between each of the seven self-reported ART adherence measures and elevated VL over time while controlling for potential socio-demographic covariates.

## Methods

### Study Setting and Recruitment

This analysis uses data from the Mzantsi Wakho study, a longitudinal cohort of ALHIV. ART-initiated adolescents aged 10–19 years at baseline were recruited from a municipality in the Eastern Cape Province in South Africa, a province with an estimated HIV prevalence of 14% [24]. Participants were recruited by identifying all adolescents initiated on ART in the area through medical records reviews in 53 ART-providing public health facilities. Participants were then traced to their communities, homes, or schools, including those who had disengaged from care or been lost to follow-up (LTFU). HIV-negative peers from neighbouring homes and some co-resident adolescents were also recruited and interviewed to minimize stigma. Baseline interviews were conducted in 2014–2015, with follow-up interviews in 2016–2017 and 2017–2018.

### Data Collection

Data was collected from two sources, a self-report questionnaire, and the extraction and linking of paper and electronic medical records. *Adolescent self-report questionnaire*: Adolescents completed a tablet-based standardised questionnaire in clinics or communities with the support of research assistants trained in working with vulnerable adolescents. The questionnaires were developed with input from a Teen Advisory Group, translated into the local language (isiXhosa), and were designed to be non-stigmatizing and engaging by including graphics, interactive games, and vignettes to introduce questions around sensitive topics. Adolescents responded to questions on their experiences at home, in their communities, and in healthcare settings, as well as self-reported adherence assessed using multiple measures. Before the interview, trained community-based research assistants sat with adolescents to demonstrate how to use the tablet properly and guide them when necessary. Participants then completed the questionnaire on their own in either English or isiXhosa depending on their preference and lasted between 60 and 90 min. For the current analyses, self-reported survey data were only used to determine participants’ sociodemographic characteristics and adherence measurements. Further study information, including study protocol, is available at www.mzantsiwakho.org.za.

*Medical Records Review* at each of the 53 healthcare facilities, routine medical records (paper-based and electronic) were searched for every study participant aged 10–19 who had ever initiated ART treatment. This approach enabled the extraction of participants’ records from all included facilities where they may have received care, including undocumented transfers to a new facility. HIV-related data were extracted in two rounds using a standardised form, covering records from 2014 to 2017. This data was later supplemented by routine laboratory test data (2014–2019) from the National Health Laboratory Services (NHLS) data warehouse. The NHLS archives all routine laboratory data from public-sector health facilities in South Africa and allowed the inclusion of laboratory tests from facilities outside the study catchment area. Demographic information (name, surname, sex, and date of birth) for adolescents in the cohort was used to link to laboratory test records from the NHLS data warehouse to study participants.

### Measures

*Self-reported adherence* was measured using seven measures, as summarised in Table 1. The exact questions related to each measure are shown in Supplementary Tables 1, of which five items were adapted from the Patient Medication Adherence Questionnaire [21]. The weekend [25] and the clinic appointment measures [26] were added based on recommendations from other studies and our qualitative research team [27]. *HIV-1 RNA VL measures* were obtained from data abstracted from participants’ clinic folders and routine biomarker data from South Africa’s NHLS following the linkage of participants’ sociodemographic data to the NHLS data warehouse. Given that VL measures were taken in line with the participant’s clinic VL monitoring schedule and did not always align with their study interview dates, we assigned the closest VL result, within 12 months from the interview date. The median time of VL records date from the interview date was 2 months (interquartile range: 1, 5). Elevated VL was defined as VL ≥ 1000 copies/mL.

**Table 1:** Adherence measurement questions and coding

Measure	Question as per questionnaire	Study definition
**Missed dose measured using positive framing of pill intake**		
*Any past 3-days missed dose*	1. How many times did you take your ARVs or HIV medicine yesterday? 2. How many times did you take your ARVs or HIV medicine the day before yesterday? 3. How many times did you take your ARVs or HIV medicine three days ago? 4. How many times a day do you have to take your ARVs or HIV medicine?	Calculated the total number of times the adolescent took all their ARVs in the past three days. If the reported pill intake did not equal the expected prescribed number of pills for the three days, then we assigned them to the non-adherent group.
*Any past-week missed timing of dose*	How many days did you take all of your ARVs or HIV medicine at the right time last week?	The responses ranged from 0 to 7 days. Missed dose timing any was defined as a binary indicator for missing dose timing (did not take their ARVs at the right time) at least one day in the past week.
*Any past-month days missed*	How many days in the last month did you want to take your ARVs but you couldn’t?	The responses ranged from 0 to 31 days. Missed dose days any was defined as a binary indicator for failing to take ARVs for at least one day in the last month.
**Missed dose measure using negative framing of pill intake**		
*Any weekend missed dose*	How many times did you not take your medication last weekend (Friday night, Saturday, and Sunday)?	Defined as 1 if the participant did not take their medication at least once in the last weekend and 0 otherwise.
*Any past-week missed dose*	Did you miss taking any of your ARV pills or HIV medicine in the last week?	Defined as a binary indicator of missing ARV pills at least once in the last week.
*Any past-month missed dose*	Did you miss taking any of your ARV pills or HIV medicine in the last month?	Defined as a binary indicator of participants missing ARV pills at least once in the last month.
**Missed dose measure as delayed refill**		
*Any past-year missed clinic appointment*	How many times in the last year were you not able to get to your clinic appointment?	Dichotomised to 1 if the participant reported missing clinic appointments at least once in the last year and 0 otherwise.

*All adherence items are dichotomised and negatively coded to represent non-adherence

*Socio-demographic characteristics* included age, sex, urban/rural location, living in formal or informal housing- (based on whether the adolescent reported living in an informal house-shack), and an indicator of household poverty [3, 28]. Poverty was defined as lacking access to any of the following eight basic necessities: food, clothing, doctor, fees, shoes, toiletries, uniforms, and school equipment. These items were selected as necessities by over 80% of respondents in a nationally representative South African survey [29]. The socio-demographic factors in this study were selected based on evidence from previous systematic reviews and qualitative studies on factors associated with ART adherence in this population group [3, 28, 30]. HIV care factors included time of ART treatment (years) and mode of HIV acquisition. Mode of HIV acquisition was determined following existing SSA paediatric cohorts: age of ART initiation cut-off (≤ 10 years) [31–33].

### Statistical Analysis

This analysis was restricted to participants who completed the questionnaire at all three time points. First, we assessed if there were sociodemographic and clinical differences between adolescents who completed the questionnaire at all three study rounds and those who missed at least one, and between adolescents with VL records and those without, using the t- and chi-square tests. Second, we summarised the characteristics of all participants included in the analysis at all three time points, including self-reported adherence measures (levels of non-adherence by measure) and HIV care factors. Then, using Cronbach’s alpha and item correlation, we assessed how closely related the seven self-report ART adherence items are, as a group, in measuring the same concept [34, 35]. Third, we fitted unadjusted and adjusted random effects models to assess the association between each self-reported measure and elevated VL (≥ 1000 copies/mL). The random-effects logistic regression model was used, to utilise the repeated measures structure of the data (data from the same subjects at three-time points) as well as to be able to include time-invariant factors like sex and mode of HIV acquisition. Lastly, we assessed the accuracy of each of the self-reported measures in confirming elevated VL, by computing different measures of test accuracy –sensitivity, specificity, positive predictive value (PPV), negative predictive value (NPV), and area under the receiver operating characteristic (ROC) curve, AUROC. We then explored the benefit of using one or more adherence measures to assess non-adherence. To achieve this, we combined the three best performing measures significantly associated with elevated VL, to assess if they improved the ability to predict elevated VL relative to single measures, using the measures of test accuracy. SAS v.9.4 was used to estimate measures of test accuracy using repeated measures data [36, 37]. For the rest of the analysis, we used Stata v.16.0 (Stata Corporation, College Station, Texas, USA).

We conducted a sensitivity analysis to assess the impact of missingness in the VL load measure on the relationship between each adherence measure and elevated VL using missing data imputation models. Multiple imputations by chained equations were used to impute missing VL values and the multivariable random-effects logistic regression models were applied to 20 imputed data sets, and results were combined using Rubin’s rules for each model [38].

### Ethical Approval

Ethical Approval for the Mzantsi Wakho study was granted by the University of Cape Town (UCT/CSSR/2013/4 and UCT/CSSR/2019/01), Oxford University (CUREC2/12–21), provincial Departments of Health and Education, NHLS Academic Affairs and Research Management System (2019/08/07) and ethical review boards of participating healthcare facilities. At all study waves, adolescent participants and their caregivers provided voluntary, informed, and written consent for participation, including interviews and access to adolescents’ clinical records. In cases of low literacy among adolescents or caregivers, all information and consent procedures were read aloud in the participant’s preferred language. There were no financial incentives for study participation, but all participants received a certificate of participation, snacks, and a small gift pack, including pencils and soap. Adolescents who refused to participate were still given snacks.

## Results

### Participant Characteristics and HIV Outcomes

A total of 1046 ALHIV completed the questionnaire at baseline and the study had a 90% uptake with 94% retained at Wave 2, 97% at Wave 3, and 35 (3.4%) were ascertained to have died at the end of the study. 933 (89.2%) adolescents completed the questionnaire at all three waves and were included in this analysis. Overall, there were no significant differences in baseline characteristics of participants excluded in the analysis (lost-to-study follow-up) and those retained (complete), other than that those excluded were likely to be older (Supplementary Table 2).

The descriptive summary of adolescents retained in the analysis is shown in Table 2. The majority of the sample were females (55.1%) with a mean age of 13.6 years (SD = 2.9) at baseline. Overall, the proportions residing in rural areas and informal housing structures were similar across the study waves, and those who lacked any of the 8 basic necessities (categorised as living in poverty) ranged between 67 and 78% during the study period. About 786 (84.2%) adolescents had at least one VL result at any time point, and slightly over 50% had a VL result at each time point. Adolescents without VL across the study period were also more likely to be older, live in formal housing, and have a shorter time on ART (Supplementary Table 3). Among those with any VL, about 300 (35.5%) had at least one elevated VL at any of the three waves of data and VL non-suppression rates increased from 20% at baseline to about 28.7% at Wave 3. In terms of self-reported ART adherence measures, the proportions reporting non-adherence ranged between 15 and 23% over the three waves for most measures except –*any past-week missed timing of dose* and –*any past-month missed dose* with as high as 44.3% and 32.3% respectively.

**Table 2:** Socio-demographic characteristics, self-reported ART non-adherence, and HIV care measures of the analytic sample (N = 933)

	Baseline	Wave 2	Wave 3
Measures	n (%)	n (%)	n (%)
**Socio-demographic characteristics**			
Age (Mean/SD)	13.56 (2.88)	15.07 (2.88)	16.26 (2.90)
Rural	249 (26.7)	230 (24.7)	223 (23.9)
Informal housing	172 (18.5)	134 (14.4)	131 (14.1)
Poverty	633 (67.8)	726 (77.8)	630 (67.5)
**HIV care**			
Recently acquired HIV*	197 (21.3)	197 (21.3)	197 (21.3)
Time on treatment (in years)- (Mean/SD)	4.46 (3.21)	6.00 (3.46)	7.19 (3.51)
Any viral load result (VL)	574 (61.5)	477 (50.1)	498 (53.4)
Elevated VL (**≥** 1000 copies/mL) ( n1 = 574, n2 = 477 n3 = 498)^€^	114 (19.9)	104 (21.8)	143 (28.7)
**Self-reported ART adherence measures**			
Any past 3-days missed dose	135 (14.5)	163 (17.5)	130 (13.9)
Any past-week missed timing of dose	201 (21.5)	412 (44.3)	264 (28.4)
Any past-month days missed	202 (21.7)	159 (17.1)	142 (15.3)
Any weekend missed dose	214 (23.0)	173 (18.6)	142 (15.3)
Any past-week missed dose	204 (21.9)	193 (20.7)	148 (15.9)
Any past-month missed dose^x^	301 (32.3)	246 (26.4)	182 (19.5)
Any past-year missed clinic appointment	164 (17.6)	167 (18.7)	135 (14.7)

^x^11 participants missing for this variable at Wave 2 and 3; ^€^n1, n2, and n3 represent the total number of participants with VL at each wave, respectively; *Based on the mode of HIV acquisition variable.

### Adherence Measures Characteristics

As a group, the seven measures showed high internal consistency and inter-item correlation (Table 3). The average inter-item correlation for the test scale was 0.423, which is within the recommended range of 0.15–0.60 [39], showing that the measures are well correlated. Similarly, Cronbach’s alpha (α) coefficient for the set of measures as a group was 0.837 at baseline, which is above the recommended 0.70 level, suggesting that the measures align well together and measure the same construct. Similar levels of internal consistency and item correlation were observed in Wave 2 and 3 (Supplementary Table 4).

**Table 3:** Summary of adherence measures characteristics using baseline data (Cronbach’s alpha)

	Baseline			
Item	Item-test correlation	Item-rest correlation	Average inter-item correlation	Alpha(α)
Any past 3-days missed dose	0.602	0.449	0.459	0.836
Any past-week missed timing of dose	0.792	0.695	0.396	0.797
Any past-month days missed	0.697	0.569	0.392	0.795
Any weekend missed dose	0.663	0.525	0.454	0.833
Any past-week missed dose	0.801	0.708	0.392	0.794
Any past-month missed dose	0.804	0.711	0.427	0.817
Any past-year missed clinic appointment	0.616	0.466	0.438	0.824
**Test scale**			**0.423**	**0.837**

*α- Cronbach’s alpha

### Relationship Between self-reported ART Adherence Measures and Elevated VL

Five out of the seven self-reported ART adherence measures were significantly associated with elevated VL in both unadjusted models and covariate-adjusted models (Table 4). In the covariate-adjusted models, elevated VL was significantly associated with non-adherence measured as *any missed dose –past 3-days* (aOR 3.63, 95% CI 2.06–6.39), *–past week* (aOR 1.97, 95% CI 1.18–3.29), *–past month* (aOR 1.95, 95% CI 1.22–3.12), *any past-month days missed* (aOR 1.87, 95% CI 1.11–3.13), and *any missed clinic appointment* (aOR 2.45, 95% CI 1.39–4.32). The AUROC for all the covariate-adjusted models ranged between 64.0 and 66.2%. A sensitivity analysis assessing the impact of missingness in the VL load measure on the relationship between each adherence measure and elevated VL showed similar results (Supplementary Table 5).

**Table 4:** Random-effects models showing the association between self-reported ART adherence measures and elevated VL (≥ 1000 copies/mL)

	Unadjusted models		Adjusted models		AUROC (%)
Adherence measures	OR (95% CI)	p-value	aOR (95% CI)	p-value	
Any past 3-days missed dose	3.79 (2.13–6.74)	**< 0.001**	3.63 (2.06–6.39)	**< 0.001**	66.2 (63.1–69.3)
Any past-week missed timing of dose	1.52 (1.00-2.30)	**0.048**	1.40 (0.93–2.11)	0.107	64.0 (60.9–67.2)
Any past-month days missed	2.13 (1.25–3.62)	**0.005**	1.87 (1.11–3.13)	**0.019**	64.5 (61.3–67.7)
Any weekend missed dose	1.77 (1.06–2.96)	**0.029**	1.65 (0.98–2.72)	0.051	64.4 (61.2–67.5)
Any past-week missed dose	2.12 (1.27–3.56)	**0.004**	1.97 (1.18–3.29)	**0.009**	64.4 (61.2–67.6)
Any past-month missed dose	2.11 (1.31–3.39)	**0.002**	1.95 (1.22–3.12)	**0.005**	64.5 (61.4–67.7)
Any past-year missed clinic appointment	2.63 (1.49–4.64)	**0.001**	2.45 (1.39–4.32)	**0.002**	64.9 (61.8–68.1)

^¥^The adjusted model controls for the following factors: adolescent age, sex, rural residence, informal housing, poverty, study wave, time on treatment, and mode of HIV acquisition. 95% CI–confidence interval; Age and time on treatment were significantly associated with elevated viral load across all the seven adjusted models. aOR: adjusted odds ratio. AUROC-area under the receiver operating characteristic curve for the covariate-adjusted models

### Predictive Validity of self-reported ART Adherence Measures

Sensitivity, specificity, PPV, and NPV of using self-reported adherence measures to predict elevated VL (**≥** 1000 copies/mL) are summarised in Table 5 and Supplementary Fig. 1. In this study, we sought to maximize sensitivity and PPV since the focus is on identifying adolescents who are non-adherent and, therefore, likely to have elevated VL. Overall, all seven adherence measures had a high sensitivity, suggesting that having an elevated VL was mostly related to non-adherence to ART. The PPV values of all the adherence measures were fairly high (over 77%), suggesting a higher chance that adolescents who are non-adherent have an elevated VL. The *past 3-days missed dose* measure performed best compared to other measures in predicting elevated VL with relatively high sensitivity: 91.6% (90.3–92.8) and positive predictive value: 78.8% (77.2–80.4), followed by *any past-year missed clinic appointment* with a sensitivity of 88.3% (86.8–89.8) and a positive predictive value: 78.4% (76.8–79.9).

**Table 5:** Sensitivity, specificity, PPV, and NPV for predicting elevated VL (≥ 1000 copies/mL)

Adherence measure	Sensitivity (%)	Specificity (%)	PPV (%)	NPV (%)
Any past 3-days missed dose	91.6 (90.3–92.8)	18.8 (15.2–22.4)	78.8 (77.2–80.4)	40.5 (34.6–46.3)
Any past-week missed timing of dose	74.2 (71.9–76.5)	30.8 (26.6–34.9)	77.9 (76.0-79.8)	26.6 (23.3–29.9)
Any past-month days missed	86.2 (84.4–87.7)	20.0 (16.4–23.6)	78.0 (76.3–79.7)	30.3 (25.5–34.9)
Any weekend missed dose	87.0 (85.4–88.6)	20.2 (16.6–23.8)	78.2 (76.5–79.9)	32.2 (27.3–36.9)
Any past-week missed dose	86.7 (85.1–88.3)	19.1 (15.7–22.5)	77.9 (76.2–79.6)	30.4 (25.7–35.1)
Any past-month missed dose	79.5 (77.5–81.4)	27.2 (23.2–31.1)	78.2 (76.4–79.9)	28.7 (24.9–32.4)
Any past-year missed clinic appointment	88.3 (86.8–89.8)	19.9 (16.6–23.3)	78.4 (76.8–79.9)	34.1 (29.2–39.1)

Sensitivity is the proportion of adolescents with elevated VL who are identified by non-adherence; specificity is the proportion of adolescents with suppressed viral load who are identified by adherence; PPV (positive predictive value) is the probability of adolescents who are non-adherent having an elevated VL; NPV (negative predictive value) is the probability of adherent adolescents having suppressed VL. 95%CI –confidence interval in parentheses

### Accuracy of Combined self-reported ART Adherence Measures

Table 6 illustrates the change in accuracy parameters for single compared to combined self-reported ART adherence measures in predicting elevated VL based on the items significantly associated with elevated VL in Table 5. For example, based on results in Tables 4 and 5, combining the *past 3-days missed dose* measure with the next two best measures (*any past-year missed clinic appointment and past-week missed dose)* incrementally, improved sensitivity by approximately 5%, from 91.6% with a single best measure to 96.4% with all top three measures combined while PPV remained stable at around 78%.

**Table 6:** Sensitivity, specificity, PPV, and NPV parameters for combined versus single adherence measures in predicting elevated VL (≥ 1000 copies/mL) among ALHIV

Adherence measures (combination)	Any past 3-days missed dose	Any past 3-days missed dose AND Any past-year missed clinic appointment	Any (past 3-days AND past-week) missed dose AND Any past-year missed clinic appointment
**Parameter**			
Sensitivity (%)	91.6 (90.3–92.8)	95.8 (94.9–96.6)	96.4 (95.6–97.1)
Specificity (%)	18.8 (15.2–22.4)	9.7 (7.1–12.3)	8.0 (5.8–10.3)
PPV (%)	78.8 (77.2–80.4)	77.7 (76.2–79.3)	77.5 (76.0-79.1)
NPV (%)	40.5 (34.6–46.3)	41.2 (33.2–49.2)	40.3 (31.9–48.6)

^¥^95% CI–confidence interval in parentheses.

## Discussion

To the best of our knowledge, this study is one of the first efforts to empirically investigate the longitudinal association of multiple self-reported ART adherence measures with elevated VL using ALHIV data from South Africa. This analysis had four main findings. First, all seven measures assessed among ALHIV demonstrated good psychometric characteristics. Second, five of the seven self-reported adherence measures: *any missed dose –past 3-days*, *–past week*, *–past month*, *past-month days missed*, and *missed clinic appointment*, were significantly associated with elevated VL in both univariable and multivariable models and had the best ability to predict viral non-suppression. In contrast, *missed timing of doses in the past week* and *past weekend missed doses* were not significantly predictive of elevated VL. Third, the *past 3-days missed dose* measure performed best in predicting elevated VL compared to other measures. Fourth, a combination of self-reported adherence measures maximised sensitivity in predicting elevated VL compared to single measures alone.

Self-reported measures range from single items on missed doses in a specified time to more complex items requiring a detailed recall. Five of the seven self-reported measures assessed in this study (i.e., *any missed dose* in the *past 3-days*, *–past week*, *–past month*, as well as *past-month days missed*, and *missed clinic appointment)* were significantly associated with elevated VL even after adjusting for potential confounders. Although few studies report using the same self-reported adherence measure which makes it difficult to compare results across studies [1, 18], these findings are consistent with previous studies from LMICs that also show that self-reported adherence measures were able to predict detectable VL [5, 22, 23, 25]. An early study in the US validating self-reported measures based on doses taken in the past month, past Saturday, and past non-weekend day among ALHIV found significantly lower VL among those who were adherent based on all measures [40]. Similarly, another study in Zimbabwe assessing self-reported adherence measures among older children and adolescents found that missed doses in the past three days, weekends, and three months, were strong indicators of elevated VL [22]. More recent studies among adolescents in Cameroon [23] and in Uganda [41] using a single item on missed doses in the past month, also found an that past month was predictive of VL. These studies used measures with varying recall periods ranging from one day to three months, similar to those used in our analysis.

An important finding is that corresponding high sensitivity (over 75%), high PPV (above 77%), low specificity, and sub-optimal adjusted AUROC (slightly above 64%) were detected in this study for all the measures. The high sensitivity observed in this study suggested that non-adherence leads to elevated VL, while the low specificity suggested that good adherence was not the only factor that can be accountable for viral suppression. This is mirrored by the low AUROC obtained in this analysis (below 0.70, the minimal value for screening purposes) even after adjusting for potential confounders. Previous research has shown that, other than poor adherence to ART, factors such as viral susceptibility, drug resistance, drug interactions, the potency of the regimen, and host immunological status may also influence one’s virological response [42, 43]. Although global recommendations are moving towards making VL monitoring the standard of care for ART programmes, in reality, there are still gaps in access in resource-limited settings due to logistical and financial constraints. Given the infrequent VL testing and potential delays in the feedback of results in many resource-limited settings, the adherence measures identified in this study may facilitate interim adherence assessments to allow for rapid assessment of adherence risk, and immediate feedback and counselling, particularly in this vulnerable group [22]. Our findings suggest that these simple and low-cost self-report measures may be valuable for both research and alternative models of care and support for adolescents who may benefit from adherence counselling and intervention [44].

The *past 3 days missed dose* measure performed best in predicting elevated VL compared to other measures. Previous studies, mostly among adults living with HIV, have had mixed findings on this measure, for example, a cross-sectional study evaluating self-reported adherence measures among (N = 2146) participants in China found that the past one-month doses taken measure might have similar accuracy compared with the 3-day measure as both were statistically significantly associated with detectable VL [25]. In contrast, another study in an ART-naïve cohort (N = 230) of adults and adolescents (≥ 12 years) in South Africa found that although a 3-day self-report yielded the highest adherence, it was not a significant predictor of viral suppression [45]. This is similar to the findings in a study among 156 participants in the U.S., which found that the 3-day recall period did not perform better than longer time periods [46]. Previous research demonstrates that short-term self-reported measures may overestimate adherence due to recall and social desirability bias [18, 45, 46]. However, the measures used in our study, including the 3-day self-report, were assessed by lay community staff, trained to be sympathetic and kind to adolescents, thereby reducing the risk of social desirability bias. Therefore, our findings maybe suggest that following careful and adolescent-sensitive interviewing, shorter recall assessments may be better suited to predict elevated VL among ALHIV. For increased generalisability, further studies could look into the applicability of these measures to routine clinical settings in high-volume ART clinics.

Furthermore, our study showed that combining different self-reported measures of adherence results in higher sensitivity and PPV for predicting elevated VL, which may be useful in clinical and research settings. These results suggest that researchers and clinicians may use multiple self-reported measures of adherence to obtain a more comprehensive assessment of adherence, resulting in a better prediction of virologic failure [45]. This may also mitigate the ceiling effect of reportedly perfect adherence, associated with self-reported measures [18]. Further research on these combinations may help develop standardised adherence measurement tools with combined measures to be used in clinics and research.

Self-report measures are relatively easy to administer and can be an opening prompt for further discussion between a patient and their provider or peer supporter to address non-adherence. The measures used in this study are a combination of shorter recall timeframes (past 3 days) which may be less susceptible to recall bias and longer timeframes (past month) which may also capture the variation in adherence behaviours, which makes them more relevant for first-stage adherence screening. The clinic appointment measure may also be useful to peer supporters as a good indicator of non-adherence risk for adolescents transitioning or moving into adherence community support groups. In general, our findings suggest that researchers, clinicians, and other forms of care may continue to use one or multiple self-reported adherence measures with some confidence in their validity at least in terms of their associations with elevated VL, as assessment tools to facilitate subsequent VL testing and support for adolescents.

This study found no significant association between *missed timing of doses in the past week*, or *past weekend missed dose* and elevated VL. The finding of *missed timing of doses in the past week* not being predictive of elevated VL may be because newer ART regimens are more potent and forgiving of dose timing compared to the older regimen [47], although sticking timing of dosage is still recommended, as it helps fit medication-taking into routines. The lack of association between elevated VL and *any missed dose (weekend)* measure could be partly related to Wilson et al.’s argument that asking individuals who are unintentionally non-adherent about missed doses may increase the risk of reporting intention instead of action [48]. It is also possible that participants’ responses to *any missed dose (weekend)* question was biased towards under-reporting their poor adherence behaviour since the two-and-half-days (Friday night, Saturday, and Sunday) were combined in one question.

Our study is not without limitations. First, we use self-reported adherence measures which are prone to social desirability bias and recall bias as well as question misinterpretation [18, 20, 22, 48, 49]. However, the questionnaire was administered by research interviewers outside of the routine ART care service and in the absence of the time constraints associated with routine ART clinics, and who were trained to work with adolescents reducing the risk of social desirability bias. Second, this cohort had 3.4% mortality, with a risk that those adolescents who died were more vulnerable, again potentially risking underestimation of effects. Third, our VL measure was missing for a number of participants, and 15.8% of the analytic sample had no VL measure at all three waves. This may have underestimated the extent of virological treatment failure. To address this, we assessed predictors of missing VL and fitted missing data imputation models to model the impact of missingness in the relationship between self-reported adherence measures and VL. Fourth, VL data used in this analysis did not match the questionnaire dates exactly but was within 12 months from the questionnaire dates which may bias the relationship between adherence and elevated VL. The strength of this study is that it is a longitudinal study that included multiple self-reported adherence items with varying item content and recall timeframes among ALHIV. The self-reported data is also based on standardised questionnaires, administered through a study that actively traced adolescents over multiple waves, allowing the inclusion of adolescents who have moved between health care facilities or disengaged from care. This study also provides evidence from a sample of adolescents initiated on ART through government services in over 53 clinics in South Africa’s Eastern Cape Province, a province affected by poor health infrastructure and high rates of HIV [24]. Therefore, these findings may be generalisable to other countries or similar contexts in sub-Saharan Africa.

## Conclusions

In summary, our study shows that self-reported adherence measures may be used to screen for non-adherence and potentially flag current or pending elevated VL in ALHIV. Predictive validity could potentially be improved by combining multiple self-reported measures. Adherence monitoring during adolescence requires more attention in the era of universal ART for all, therefore, we recommend that clinicians and researchers continue using self-report questions (single or in combination) in their day-to-day clinical care or research practice to detect non-adherence. These findings facilitate the development of low-cost and relatively easy-to-administer self-reported adherence tools for use among adolescents in low-resource settings.

### Electronic Supplementary Material

Below is the link to the electronic supplementary material.

Supplementary Material 1

## Data Availability

Data is available upon request following correct procedures (for more information please see: https://www.mzantsiwakho.org.za/publications). Data is not yet available open access as the study has not concluded, therefore data is not fully anonymised.

## Code Availability

STATA do-files are available upon request from co-authors.
