# Supplementary material for: Exploring Self-reported Adherence Measures to Screen for Elevated HIV Viral Load in Adolescents: A South African Cohort Study
Source: AIDS Behav. 2023 Apr 17;27(11):3537–47. doi: 10.1007/s10461-023-04068-2 (PMC10589188; doi:10.1007/s10461-023-04068-2)
Supplement: Supplementary file 1 — Supplementary Material 1 [file 10461_2023_4068_MOESM1_ESM.docx]

**Exploring self-reported adherence measures to screen for elevated HIV viral load in adolescents: A South African Cohort Study –Supplementary material**

Table of contents

**Table 1.** Questionnaire original adherence items or measures….……………………….…….2

**Table 2.** Comparison of baseline characteristics for complete cases and LFTU^¥^ (N=1046)….3

**Table 3.** Comparison of baseline characteristics for having any VL and missing VL

measurements at all three-time points (N=933)…………………………………….3

**Table 4.** Summary of adherence items characteristics (Cronbach’s alpha)……………….......4

**Table 5.** Multilevel model odds ratios for predicting elevated VL (>=1000 copies/mL) by

different adherence measures after missing data imputation……………………..…5

**Figure 1.** Prediction of elevated viral load by each self-reported ART adherence

measure…………………………………………………………………………..…6

**Table 1: Questionnaire original adherence items or measures**

| 1. **Any missed dose (past 3 days)**   *“Sometimes people get busy and forget to take their ARVs or HIV medicine. We would like to understand if you have similar experiences when you take your ARVs or HIV medicine so we can help other teens. We need to know what is really happening, not what you think we want to hear. Nobody will get angry at you and your answers. Please be honest.”*  Please answer each question with a number:   1. How many times did you take your ARVs or HIV medicine yesterday? 2. How many times did you take your ARVs or HIV medicine the day before yesterday? 3. How many times did you take your ARVs or HIV medicine three days ago? 4. How many times a day do you have to take your ARVs or HIV medicine? |
| --- |
| 1. **Missed dose timing any (Past week)**   *“Sometimes unexpected things get in the way and prevent people from taking their ARVs or HIV medicine at the same time. Some days, people do not take their ARVs or HIV medicine at all or don't take them at the right time. This is not their fault. We would like to ask you about the times you were not able to take your ARVs or HIV medicine. We simply want to better understand why teens have a hard time taking their ARVS or HIV medicine.”*   1. How many days did you take all of your ARVs or HIV medicine at the right time last week? |
| *“Remember, your answers to our questions will help us understand what makes it easier and harder to take pills or medicine. Please answer as truthfully as you can. Your answers are completely confidential, and you will not be judged.”*   1. **Last missed dose any (past week)** 2. Did you miss taking any of your ARV pills or HIV medicine in the last week? |
| *“Remember, your answers to our questions will help us understand what makes it easier and harder to take pills or medicine. Please answer as truthfully as you can. Your answers are completely confidential, and you will not be judged.”*   1. **Last** **missed dose any (past month*)*** 2. Did you miss taking any of your ARV pills or HIV medicine in the last month? |
| 1. **Past month days missed** 2. How many days in the last month did you want to take ARVs or HIV medicine, but you couldn’t? |
| 1. **Any missed dose (weekend)**   *“On weekends, Andiwe spends time with friends and family. Sometimes he travels to visit family members or stays out late with his friends. Some weekends he stays at home, goes to church, and helps his parents and grandparents. It is not always easy for him to take his medication during Saturdays and Sundays, but he does his best. Think about last weekend – Saturday and Sunday.”*  a) How many times did you not take your medication last weekend (Friday night, Saturday, and Sunday)? |
| **7. Any missed clinic appointment**  a) In the last year, were you always able to get your clinic appointment? If ‘No’  b) How many times in the last year were you not able to get to your clinic appointment? |

**Table 2: Comparison of baseline characteristics for complete cases and LFTU^¥^ (N=1046)**

|  | **Total**  **(N=1046)** | **Complete cases (N=933)** | **Lost-to-study follow-up (N=113)** |  |  |
| --- | --- | --- | --- | --- | --- |
| **Baseline characteristics** | **N (%)** | **N (%)** | **N (%)** | **Test statistic** | **p-value** |
| Age (Mean/SD) | 13.67 (2.89) | 13.56 (2.88) | 14.57 (2.88) | -3.5 | **<0.001** |
| Female | 576 (55.1) | 514 (55.1) | 62 (54.9) | 0 | 0.964 |
| Rural residence | 272 (26.0) | 249 (26.7) | 23 (20.4) | 2.1 | 0.147 |
| Informal housing | 196 (18.7) | 172 (18.5) | 24 (21.2) | 0.5 | 0.474 |
| Poverty | 708 (67.7) | 633 (67.8) | 75 (66.4) | 0.1 | 0.752 |
| Sexually acquired HIV | 222 (21.2) | 197 (21.3) | 25 (22.9) | 0.2 | 0.695 |
| Time on treatment  (Mean/SD)  (Median/IQR) | 4.44 (3.24)  4 (2,7) | 4.46 (3.21)  4 (2,7) | 4.26 (3.50)  4 (1,7) | 0.6 | 0.565 |
| ^¥^LFTU represents adolescents’ lost-to-study-follow-up at least one-time point. At each successive wave, participants were actively followed up. Below is the breakdown of the (N=113) loss-to-follow up between Wave 1 and 3: (N=12) passed on between Wave 1 and 2, and (N=22) passed on between Wave 2 and 3, (N=55) were lost-to-follow-up for the following reasons between Wave 1 and Wave 2:- refusals, untraceable or avoidant. (N=24) were lost-to-follow-up between Wave 2 and Wave 3 either as refusals, untraceable or avoidant. Chi-square test used for binary baseline characteristics while two-sample t-test used for continous baseline characteristics. | | | | | |

**Table 3: Comparison of baseline characteristics for having any VL and missing VL measurements at all three-time points (N=933).**

|  | **Total**  **(N=933)** | **Any viral load record**^¥^ **(N=786)** | **Missing viral loads for all three years (N=147)** |  |  |
| --- | --- | --- | --- | --- | --- |
| **Baseline characteristics** | **N (%)** | **N (%)** | **N (%)** | **Test statistic** | **p-value** |
| Age (Mean/SD) | 13.56 (2.88) | 13.47 (2.85) | 14.07 (2.98) | -2.4 | **0.018** |
| Female | 514 (55.1) | 431 (54.8) | 83 (56.5) | 0.1 | 0.716 |
| Rural residence | 249 (26.7) | 206 (26.2) | 43 (29.3) | 0.6 | 0.444 |
| Informal housing | 172 (18.5) | 162 (19.2) | 14 (9.6) | 9.0 | **0.003** |
| Poverty | 633 (67.8) | 526 (66.9) | 107 (72.8) | 2.0 | 0.162 |
| Recently acquired HIV | 197 (21.3) | 162 (20.7) | 35 (24.6) | 1.1 | 0.295 |
| Time on treatment (in years)  (Mean/SD)  (Median/IQR) | 4.56 (3.21)  4 (2,7) | 4.61 (3.23)  4 (2,7) | 3.24 (2.84)  3 (1,6) | 3.8 | **<0.001** |

^¥^Outcome based on the most recent viral load within 12 months of the interview date at each time point. Any participants without any record at all time points were defined as missing VL. Chi-square test used for binary baseline characteristics while two-sample t-test used for continous baseline characteristics.

**Table 4: Summary of adherence items characteristics (Cronbach’s alpha)**

|  | **Wave 2** | | **Wave 3** | |
| --- | --- | --- | --- | --- |
| **Item** | **Average inter-item correlation** | **Alpha (α)** | **Average inter-item correlation** | **Alpha(α)** |
| Any past 3-days missed dose | 0.380 | 0.786 | 0.565 | 0.886 |
| Any past-week missed timing of dose | 0.426 | 0.817 | 0.578 | 0.891 |
| Any past-month days missed | 0.416 | 0.810 | 0.568 | 0.887 |
| Any weekend missed dose | 0.429 | 0.818 | 0.575 | 0.890 |
| Any past-week missed dose | 0.363 | 0.773 | 0.529 | 0.871 |
| Any past-month missed dose | 0.371 | 0.779 | 0.551 | 0.880 |
| Any past-year missed clinic appointment | 0.395 | 0.796 | 0.576 | 0.890 |
| **Test scale** | **0.397** | **0.822** | **0.563** | **0.900** |

*****α- Cronbach’s alpha

**Table 5: Multilevel model odds ratios for predicting elevated VL (>=1000 copies/mL) by different adherence measures after missing data imputation.**

|  | **Imputation models**^¥^ | |
| --- | --- | --- |
|  | **Elevated VL (>=1000 copies/mL)** | |
| **Adherence measures** | **aOR (95% CI)** | **p-value** |
| Any past 3-days missed dose | 3.21 (1.80-5.73) | **<0.001** |
| Any past-week missed timing of dose | 1.36 (0.91-2.02) | 0.129 |
| Any past-month days missed | 1.73 (1.04-2.86) | **0.034** |
| Any weekend missed dose | 1.58 (0.89-2.79) | 0.112 |
| Any past-week missed dose | 2.13 (1.14-3.99) | **0.020** |
| Any past-month missed dose | 2.14 (1.22-3.76) | **0.009** |
| Any past-year missed clinic appointment | 2.27 (1.26-4.08) | **0.007** |

^¥^Multiple imputations by chained equations were used to impute missing dichotomous elevated VL values. The imputation model included adolescent age, participant sex, mode of infection, time on ART treatment and adherence measure for each imputation model. The multivariable mixed-effects regression models were applied to 20 imputed data sets, and results were combined using Rubin's rules for each model. aOR: adjusted odds ratio; 95% CI – confidence interval.

**Figure 1: Prediction of elevated viral load by each self-reported ART adherence measure.**

| **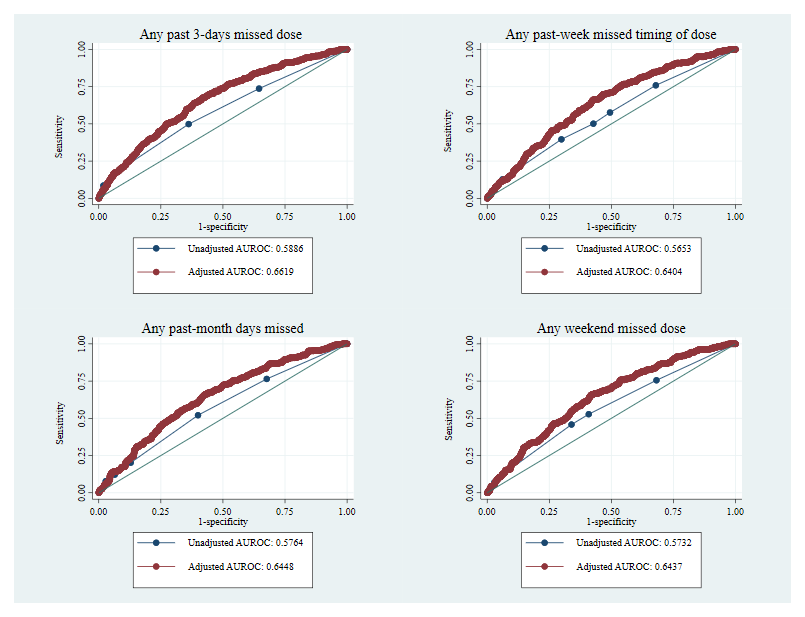** |
| --- |
| **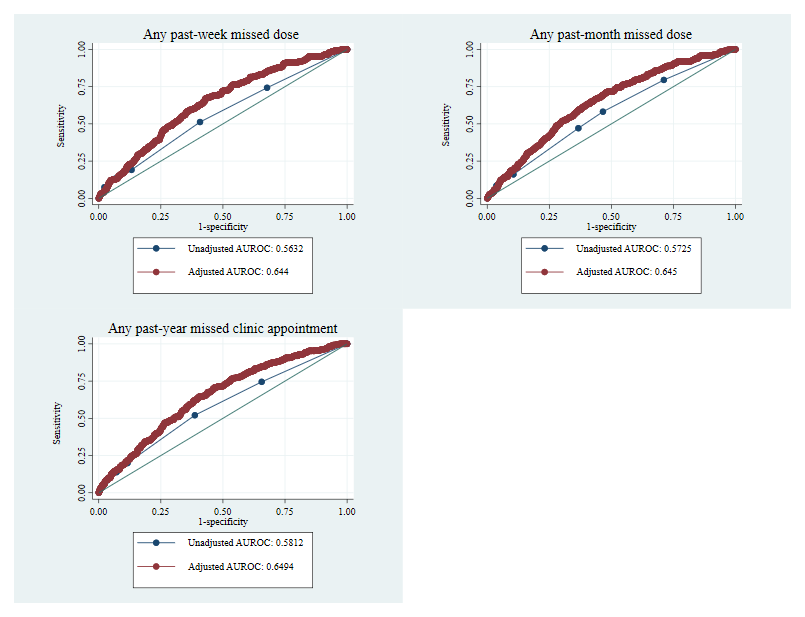** |
